# The Impact of Government Social Media Information Quality on Public Panic During the Infodemic

**DOI:** 10.3389/fpsyg.2022.908213

**Published:** 2022-05-13

**Authors:** Shanshan Zhai, Yuanxiang John Li, Maomao Chi

**Affiliations:** ^1^School of Information Management, Central China Normal University, Wuhan, China; ^2^Sawyer Business School, Suffolk University, Boston, MA, United States; ^3^Institute of Digital Commerce, Wuhan Technology and Business University, Wuhan, China

**Keywords:** infodemic, government, social media, information quality, public panic

## Abstract

The COVID-19 pandemic triggered the first global “Infodemic” in the era of social media. Understanding how governments deal with the negative impacts of the infodemic (e.g., public panic) has become a priority. This paper uses the theoretical framework of the Elaboration Likelihood Model (ELM) to explore mechanisms for alleviating panic associated with the infodemic. It considers, in particular, the quality of information circulated on Government Social Media (GSM) as the central route and local government trust as the peripheral route. An empirical study was conducted using data from a focus group interview and a questionnaire survey collected within the first three weeks following the citywide lockdown of Wuhan, China. The results show that as: (1) Quality of GSM information does not significantly reduce public panic, but local government trust significantly increases people’s pandemic prevention knowledge; (2) Pandemic prevention knowledge is a critical mediator between information quality of GSM and public panic, as well as local government trust and public panic; and (3) Information quality of GSM significantly increases people’s trust in local governments. This paper contributes to the literature on infodemic and government social media and provides implications for practice.

## Introduction

At the end of 2019, an epidemic of the novel coronavirus COVID-19 broke out in Wuhan, China. On February 11th, 2020, during a conference to discuss the virus, the World Health Organization (WHO) introduced the concept of an “infodemic” (Zarocostas, 2020). The term refers to the vast amount of COVID-19 information that quickly became available. Much of the information was of dubious quality, making it difficult to determine what should be trusted and to identify reliable sources, creating the risk of harm to the physical and mental health of the public (Lancet, 2020). This problem has been particularly acute because COVID-19 occurred in the era of social media, which has facilitated the dissemination of inaccuracies, greatly contributing to the infodemic. Because of concerns about the negative role of social media, at times of crisis, some countries have taken extreme measures. Sri Lanka, for example, responded to false reports following a terrorist incident by shutting down social media (Swisher, 2019). However, social media can be an effective educational tool. They have helped the public to learn about preventative measures during the pandemic and can be used by authorities to combat false rumors, which can help to stabilize society and public sentiment (Li and Liu, 2020). Unlike the SARS outbreaks in 2003, the Chinese government has taken full advantage of social media to release real-time pandemic information and to refute false rumors about COVID-19. Thus, it has become sensible for governments in the Omnimedia era, to consider how to make effective use of social media to alleviate the negative impact of infodemics (such as public panic). There are also some concerns about the impacts of social media on public attitudes and emotions (Oh et al., 2013; Basch et al., 2020; Ivie et al., 2020; Laato et al., 2020; Zagidullin et al., 2021; Luo et al., 2022). Some commentators argue that “too much information” will cause public anxiety, depression, and panic and may reduce the impact of positive actions taken in response to the epidemic (Laato et al., 2020; Khubchandani et al., 2021; Luo et al., 2021). For example, in an online survey of five designated COVID-19 hospitals in Hubei Province (China), Ma et al. (2020) found that patients who frequently used social media to obtain COVID-19-related information tend to be more depressed and report other negative emotions. Drawing on theories of health perception and cognitive load, Laato et al. (2020) propose that, to mitigate the COVID-19 infodemic, information overload should be reduced and suspicious health news should be screened. However, other literature suggests that by using social media, people can effectively acquire relevant public health knowledge and maintain social contact with others, which can help to reduce public anxiety (McGowan et al., 2012). For example, Basch et al. (2020) found that young people not only share important public health information but can also solve some problems (e.g., anxiety) brought by COVID-19, by using Tiktok to stay connected and positive.

However, the current literature about the impact of social media on the infodemic has two limitations. Firstly, it mainly explores the negative impact of social media: usually from the perspectives of information overload or cognitive burden. But there is also a positive role for social media in reducing the negative impacts of the infodemic, though this lacks theoretical explanations and empirical discussions. Secondly, literature about the infodemic tends to focus on the use of social media by the general public, overlooking their value to governments in the management of crises (Chen et al., 2020). Thus, it is also important to consider governmental use of social media and its role in mitigating the COVID-19 infodemic. This paper seeks to address these limitations by exploring the influencing effects of information quality on government’s social media, in particular, such as Weibo, WeChat, and Tiktok, which were created by government agencies and are used for establishing relationships with the general public (Bertot et al., 2012). According to the Elaboration Likelihood Model, a dual-process theory that describes the change in attitudes (Petty and Cacioppo, 1986, 2012); the negative effects of the infodemic (e.g., public panic) can be reduced through two influencing routes: central and peripheral. In the central route, people thoughtfully process information content that the government has posted on social media to alleviate public panic. The peripheral route relies on people believing and trusting the credibility of information sources (i.e., trusting local government) due to the association between social media and the government’s use of them to alleviate public panic. At the start of the COVID-19 pandemic, the city of Wuhan in China was closed. During the first three weeks of the lockdown (between 23 January and 13 February 2020), a survey was carried out to gauge opinion on government social media information quality, public panic, and so on. Analysis of the survey data has yielded theoretical and practical insights that help to explain how a government’s use of social media may reduce the negative impacts of an infodemic.

## Literature Review and Theoretical Background

### Research on Infodemic and Public Panic

Over the past 20 years, Information Epidemiology, as a branch of health informatics, has made developed greatly (Eysenbach, 2002, 2009; Mavragani, 2020). More recently, COVID-19 has caused the first global infodemic of the social media era (Ahmad and Murad, 2020). On February 15th, 2020, at the Munich Security Conference, the Director-General Tedros Adhanom Ghebreyesus of WHO declared as: “we are not just fighting an epidemic; we are fighting an infodemic.”^1^ Existing infodemic literature has used data from various online platforms (including social media such as Twitter and Facebook) to analyze and predict the outbreak of diseases, as well as the effectiveness of health intervention (Mavragani, 2020). These diseases can be epidemics such as drugs, marijuana, depression, and smoking (Katsuki et al., 2015; Ricard et al., 2018) or chronic diseases like diabetes and breast cancer. (Arnhold et al., 2014).

After COVID-19 began to spread, researchers began to explore whether and how the infodemic promoted by social media contributed to public panics, thereby affecting people’s physical and mental health (Ahmad and Murad, 2020). The resulting literature can be divided into two streams. One uses methods derived from information epidemiology to analyze the spread and management of the infodemic based on social media usage (Liao et al., 2020). The other analyzes impact of the use of social media on public panic, anxiety, and cyberchondria^2^ (Laato et al., 2020). For example, Ahmad and Murad (2020) conducted a survey and found that 87.01% of the panic transmission phenomenon caused by the COVID-19 can be explained by the use of social media. Laato et al. (2020) found that there is a significant positive relationship between information overload and users’ cyberchondria. Other studies found sadness, anxiety, and cognitive dissonance, resulting from the perceived threat and perceived information overload (Song et al., 2021). However, the current literature has the following two limitations. Firstly, the relevant studies in the fields of Information Epidemiology and Library and Information Science (LIS) tend to focus on the management of chronic diseases from the perspective of information overload and cognitive burden: it lacks research on the governance of infodemics. Secondly, although a few studies have explored the relationship between infodemics and public health, there is no theoretical explanation of how to reduce the negative effects of infodemic, such as public panic, anxiety, and cyberchondria.

### Research on Government’s Social Media and Crisis Management

In this paper, Government’s Social Media (GSM) refers to social media accounts (such as Weibo, Twitter, WeChat, and Tiktok) registered by the state or by local governments, to share information with the public (Bertot et al., 2012). Earlier studies have discussed this from either a government perspective or from that of the public. Studies of the latter focus on the public’s willingness to connect with the GSM. For example, using social media data from January to March 2020, Chen et al. (2020) explored the impacts of media richness, dialogue cycle, content type, and emotional value on public participation in GSM at the start of the COVID-19 pandemic. Studies of GSM from a government perspective highlight how governments use different social media platforms to release information, manage public opinion, interact with the public, and handle crises (such as emergency responses) (Zhang et al., 2019). For example, Bertot et al. (2012) explored the challenges (such as privacy protection and regulations) and opportunities faced by the US federal government in their use of social media. Kavanaugh et al. (2012) study how the government’s use of social media can help to deal with crises ranging from the frequent (such as traffic and weather crises) to the extreme (such as earthquakes and floods). When a crisis occurs, the relevant government agencies can respond by using social media to release up-to-date information, assess public attitudes and behaviors, correct false rumors, establish social cohesion, and mobilize social resources (Chatfield and Reddick, 2018; Zhang et al., 2019; Chen et al., 2020). However, despite a current growing body of research that recognizes the potential value of GSM in crisis management, the particular means by which GSM counters an infodemic have yet to be empirically explored.

### Elaboration Likelihood Model

**Figure 1:**
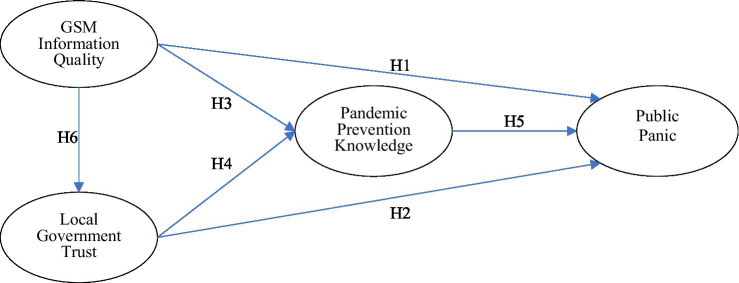
The research model.

The Elaboration Likelihood Model (ELM) was proposed by Petty and Cacioppo (1986) to describe the formation and change of attitudes toward external stimuli (e.g., information). The model considers two distinct routes of information processing and attitude formation, namely, the central route and the peripheral route (Petty and Cacioppo, 2012). The central route tends to require intentional and cognitive attention to process relevant information (such as text or verbal expression). Information processing by the peripheral route, by contrast, often evaluates quality based on an overall impression of the information (such as attractiveness, credibility, or perception of the sources) (Petty and Cacioppo, 2012). Choice of route is when processing information depends on the elaboration likelihood (Petty and Cacioppo, 1986). People who process information through the central route tend to view information rationally, examine relevant information carefully, and logically change their attitudes. Individuals who process information through the peripheral route, think less about the information itself, and instead are influenced by other factors (Sussman and Siegal, 2003; Bhattacherjee and Sanford, 2006). ELM was initially applied to offline consumer behavior, but the model has recently been used to explain consumers’ behavior on social media (Li et al., 2019; Chang et al., 2020). For example, Chang et al. (2020) used ELM to explore the impact of central route factors (information completeness and information accuracy) and peripheral route factors (post esthetics and post popularity) on consumer behaviors in Facebook’s second-hand market. In addition, Li et al. (2019) analyzed how characteristics of social media affect the influence of corporate apologies and corporate reputation on customer attitudes. Furthermore, by integrating the Technology Acceptance Model with ELM to build an information adoption model (IAM), Sussman and Siegal (2003) found information usefulness to be a mediator of the information adoption process. Originally, the ELM asserted that only one of the two routes (central or peripheral) influenced a change of attitude. However, a review of the literature suggests that both of them jointly affect the attitudes and intentions of individual behaviors (Cyr et al., 2018). This study explores the impact that the quality of GSM information has on public panic which is the outcome of public information adoption and aims to increase understanding of the mechanisms of both the central route and peripheral route causing public panic. The information quality of GSM is a high level of elaboration and represents a centrally controlled means of influencing how the public is informed. At the same time, trust in the government may act as an informational indicator that helps people assess the “argument quality” of the information provided, rather than the content itself thus representing a peripheral route. This paper extends the original ELM by exploring the mediating role of pandemic prevention knowledge in reducing panic, and it tests the effects of GSM information quality on trust in the government. Our research model is shown in Figure 1.

## Research Hypotheses

### Direct Effect of Quality of the Information in GSM and Local Government Trust on Public Panic

Information quality affects the persuasive power of relevant arguments (such as textual information) embedded in information (Delone and McLean, 2003; Bhattacherjee and Sanford, 2006) including the complete, consistent, and accurate information (Delone and McLean, 2003). The Quality of GSM Information in this study refers to the public’s evaluation of the quality of information relating to the pandemic that they obtain from the GSM. During the COVID-19 pandemic, Ahmad and Murad (2020) surveyed the Iraqi public and found a significant positive correlation between the use of social media (i.e., Facebook) and public panic. They concluded that the association arose because social media is filled with excessive information, making it hard to distinguish the true from the false. The government uses social media to transmit complete, consistent, and accurate pandemic information for public crisis management (such as releasing authoritative information and refuting rumors) (Kavanaugh et al., 2012). These actions can effectively alleviate infodemics (such as rumors of epidemics; Li et al., 2021) and the higher the quality of information circulated by GSM, the more effective it is in alleviating public panic associated with infodemics. Therefore, we propose the following hypothesis:

*Hypothesis 1*: A high level of GSM information quality negatively affects public panic (H1).

According to the ELM, source credibility refers to the perception of the quality of a message source and reflects nothing about the message itself (Sussman and Siegal, 2003; Bhattacherjee and Sanford, 2006). Source credibility of GSM is determined by the public’s perception of trust in their local government. The existing literature considers three dimensions that influence trust: government ability, benevolence, and integrity (Zahedi and Song, 2008). Government ability relates to its competence in the relevant work, such as management of human, financial, and material resources and, during the COVID-19 epidemic, pandemic prevention and control. Benevolence is determined by the extent to which a government actively cares for and helps the public, by, for example, improving social welfare. A government with integrity handles public affairs fairly, for example, distributing pandemic prevention materials fairly.

According to the Elaboration Likelihood Model, information with “source credibility” (such as information from local government) changes the public’s attitudes or behaviors by the peripheral route. In the case of a low elaboration, source credibility can directly change public attitudes or opinions (Bhattacherjee and Sanford, 2006). For example, Wang et al. (2019) found that the credibility of game providers could prevent users from cheating in an online game. Li et al. (2019) also find that the credibility of the source (company reputation) could change public attitudes during a company’s crisis. During an infodemic, the more trust the public has in their government, the more confidence society has in fighting the epidemic. Thus, this trust relationship can effectively fight the pandemic and alleviate public panic. Therefore, we propose the following hypothesis:

*Hypothesis 2*: A high level of local government trust negatively affects public panic (H2).

### Pandemic Prevention Knowledge as the Mediator in the Information Adoption Process

According to the ELM, information usefulness relates to the user’s perceptions of valuable, informative, and helpful information (Delone and McLean, 2003; Sussman and Siegal, 2003). Research by Blair et al. (2017) suggests that, during an infodemic, the usefulness of information will be strongly influenced by the public’s understanding of pandemic prevention knowledge. In China, through GSM, the public has access to information relevant to pandemic prevention. This includes details of transmission routes, symptoms, and COVID-19 preventive measures. According to the information adoption model (Sussman and Siegal, 2003), by representing the information usefulness, pandemic prevention knowledge is a critical mediator in the information adoption process. Pandemic prevention knowledge can be induced by the information quality of GSM and local government trust and further affect public panic. Where government social media provides information of high quality, awareness of COVID-19 preventive measures is increased, helping to mitigate the pandemic. Research suggests that the use of social media (e.g., Tiktok) can encourage the uptake of public health information and promote physical and mental health (Basch et al., 2020). Elsewhere, Mensah et al. (2021) found that information quality has a positive effect on the perceived usefulness of COVID-19 informational e-government services. Many governments actively disseminate information relevant to the pandemic through various social media platforms (e.g., micro-blogs, WeChat, Tiktok, and Twitter), as well as refuting false rumors and misinformation. Therefore, we propose the following hypothesis:

*Hypothesis 3*: A high level of information quality on GSM positively affects pandemic prevention knowledge (H3).

Based on Sussman and Siegal (2003)’s study of ELM, source credibility can directly affect the public’s perception of information usefulness, which refers to a crossing route effect. As a result, trust in local government trust can directly influence the transmission of knowledge relevant to preventing the pandemic. A survey carried out during an outbreak of the Ebola virus found that respondents who had low trust in their government were less likely to take relevant preventative measures to deal with the epidemic (Blair et al., 2017). This suggests that the more trust the public has in the government, the more it is willing to use the information provided by GSM and take preventive measures. Thus, we put forward the following hypothesis:

*Hypothesis 4*: A high level of local government trust has a positive impact on pandemic prevention knowledge (H4).

According to the ELM, perceived information usefulness can affect consumers’ attitudes and opinions (Sussman and Siegal, 2003). Bhattacherjee and Sanford (2006), for example, find that it would change users’ attitudes toward information technology. During the infodemic, knowledge about COVID-19 transmission routes, symptoms, and preventive measures is an important carrier of information usefulness of GSM (Han et al., 2021). If the public can understand such pandemic prevention knowledge, it is likely to reduce levels of panic or anxiety caused by the infodemic. This is because an objective understanding of the pandemic and effective screening of associated rumors help to alleviate information overload. Therefore, we propose the following hypothesis:

*Hypothesis 5*: Pandemic prevention knowledge negatively affects public panic (H5).

### The Effects of Information Quality of GSM on the Local Government Trust

By extending the ELM, this study further explores the effects of the central route on the peripheral route: The quality of information on the GSM can promote public trust in the government. In contrast to the SARS epidemic in 2003, COVID-19 occurred in the Omnimedia era. Governments can make full use of social media to release real-time pandemic information, government affairs, and government attitudes and recommendations. The information shared by the local government through social media will affect public trust in the government. Previous research also suggests that government plays a positive role on social media in building public trust and cooperation, and then effectively carrying out pandemic prevention and control measures (Larson, 2018; Depoux et al., 2020; Kadam and Atre, 2020). Therefore, the higher the quality of information circulated on GSM, the more it can promote public trust in the government. We put forward the following hypothesis:

*Hypothesis 6*: A high level of information quality of government’s social media positively affects trust in local government (H6).

## Research Methodology

### Research Context

Our research took place in the early days of the COVID-19 infodemic in Wuhan, China. We began it following the lockdown announcement on January 23rd, 2020, and used focus groups and an online survey to gather data from residents. The survey questionnaire was based on a literature review, and the focus groups were used to confirm and modify the key constructs tested by the survey. A pilot test was performed before large-scale data collection. To ensure the effectiveness and consistency of the research context, the data analyzed in this paper were collected within three weeks of the city’s lockdown announcement.

### Focus Group Interview

To help refine the survey questions, we conducted online interviews through WeChat, with five professionals who stayed in Wuhan before and after the lockdown announcement. The interview lasted around 60 min each and helped us to identify several keywords. For infodemic keywords, *“Wuhan Institute of Virology,” “The Red Cross Society of Wuhan,” “Lianhua Qingwen Capsules (to clear scourge and remove toxin, diffuse the lung and discharge heat.),” “Residential property,” “The Fangcang shelter hospitals,” “the 2019 Wuhan Military World Games,”* and *“The Wuhan Jinyintan Hospital”* were proposed. For social media keywords, *“information authenticity is difficult to distinguish,” “pandemic prevention information,” “rumor,” “Refute rumors,” “local government trust,”* and *“latest data”* were drafted. And for the keywords about the negative impact of infodemic, *“anxiety,” “insomnia,” “nervousness,” “worry,”* and *“fear”* were depicted.

The initial questionnaire of this study was formulated using insights from the above keywords and the interview content. These were used in combination with the elaboration likelihood model, the information adoption model, and the infodemic literature. The initial questionnaire of this study was formulated using insights from the above keywords and the interview content.

### Measurement

This study has four major latent variables: Information Quality of GSM, Pandemic Prevention Knowledge, Local Government Trust (which includes the three second-order variables, Ability, Benevolence, and Integrity), and Public Panic. The measurement items of these variables are either directly adopted or refined from the existing scales. To ensure the reliability and validity of the measurement due to language differences, all items are translated according to the procedures of translation and back-translation. Based on results from the focus group interview, some items were revised so that the related constructs can be accurately measured and understood by residents. Finally, we have determined six aforementioned variables including 24 measurement items in total. All of them are based on Likert scales, ranging from “1” for strongly disagree to “7” for strongly agree. Specific items are shown in Table 1. The Information Quality of GSM is adapted from Delone and McLean (2003) and Sussman and Siegal (2003) to measure the three specific quality characteristics (i.e., complete, consistent, and accurate) of the infodemic information provided by the government’s social media. Pandemic Prevention Knowledge (PPK) measures the useful information obtained by the public from the government’s social media. The measurement of this variable is adapted from the scale for the Ebola epidemic developed by Blair et al. (2017). After editing to consider the different contexts, PPK measures the public’s relevant knowledge about the COVID-19 including transmission routes, symptoms, and preventive measures. Local Government Trust (LGT) measures the public’s trust in local government. This construct adopts the previous measurement and the “reflective-reflective” second-order model (i.e., the first and second-order models are both reflective) to evaluate the public’s trust in government (McKnight et al., 2002; Zahedi and Song, 2008). Specifically, the three first-order variables are Ability, Benevolence, and Integrity. Finally, Public Panic is the negative emotional impact of the infodemic on the public. Referring to the death anxiety scale created by Belmi and Pfeffer (2016), this paper uses the frequency of respondents’ recent negative emotions (i.e., scared, uneasy, anxious, and nervous) to measure public panic.

**Table 1:** Reliability and validity.

Construct	ID	Item	Loading	AVE	Cronbach’s α	C.R.
Information quality of GSM (IQGSM)	The relevant information about COVID-19 (Tiktok, WeChat, and Twitter) by using GSM					
	GSM1	Complete	0.905	0.867	0.923	0.951
	GSM2	Consistent	0.941			
	GSM3	Accurate	0.947			
Pandemic prevention knowledge (KW)	About the COVID-19:					
	KW1	I know the novel coronavirus transmission route.	0.928	0.870	0.925	0.953
	KW2	I know the novel coronavirus symptoms.	0.942			
	KW3	I know novel coronavirus prevention measures.	0.929			
Ability (GA)	After the outbreak of COVID-19, I think the local government departments:					
	GA1	…have much knowledge about the work that needs to be done.	0.891	0.835	0.951	0.962
	GA2	…have specialized capabilities that can help our citizens.	0.917			
	GA3	… are well qualified.	0.904			
	GA4	… are very capable of performing their tasks.	0.921			
	GA5	…seem to be successful in the activities they undertake.	0.935			
Benevolence (GB)	After the outbreak of COVID-19, I think the local government departments:					
	GB1	…concerned about the welfare of the citizens.	0.937	0.882	0.968	0.955
	GB2	…concerned about what citizens think is important.	0.950			
	GB3	…concerned about the needs of the citizens.	0.950			
	GB4	…will do their best to help the citizens.	0.918			
Integrity (GI)	After the outbreak of COVID-19, I think the local government departments:					
	GI1	…treat everyone as fairly as possible.	0.890	0.860	0.959	0.969
	GI2	…have a strong sense of commitment.	0.939			
	GI3	…acting on reasonable principles.	0.934			
	GI4	…have a commitment.	0.950			
	GI5	…words and deeds are consistent.	0.923			
Public Panic (PP)	In the recent, the frequency of emotions was as follows:					
	PP1	I feel scared.	0.864	0.759	0.895	0.926
	PP2	I feel uneasy.	0.911			
	PP3	I feel anxious.	0.851			
	PP4	I feel nervous.	0.859			

*All factor loading reached the significant level at 0.001 level*.

### Data Collection

After designing the questionnaire, we ran a pilot test. The initial version of the questionnaire was sent to 15 residents of Wuhan before and after the lockdown. Recipients were also asked their opinions of the questionnaire, to improve it. According to feedback received, and the results of preliminary exploratory factor analysis, the questionnaire was further revised. After completing the focus group and the pilot test, we used WeChat to launch the survey in Wuhan at the beginning of 2020. To encourage people to fill out the questionnaire, we offered small prizes set randomly between ¥3 and ¥6 (approximately $0.5–$1.0) to anyone who completed a valid questionnaire. By this means, through snowball sampling, we circulated the survey to 1,558 Wuhan residents (identified by their IP addresses), of whom 235 (15%) responded. However, nine of these were deleted based on time spent completing the questionnaire and on responses to attention-detection questions. This resulted in 226 valid questionnaires.

We also tested for non-response bias and common method bias. To test for non-response bias, we compared data collected on the first day with data collected on the last day of collection. The analysis found no significant differences in gender, age, and educational background between those two groups (*p* > 0.1). Secondly, the main variables in this study were tested from the same respondents, which may induce common method variance (CMV) (Podsakoff et al., 2003). To check this, we tested Harman’s single factor and Marker variables. The results of Harman’s factor test showed that the first factor accounts for 35.21% of all explanatory variables, which is less than the threshold (50%) (Chi et al., 2020). Meanwhile, the Marker variable (the cultural dimension of individualism/collectivism in the questionnaire) and the partial correlations of each variable were calculated as a conservative estimate of CMV (Lindell and Whitney, 2001). All of the between item correlations were adjusted by partitioning out the CMV estimate. The resulting changes in correlation were slight, with no change in significance, suggesting that CMV in this paper is unlikely to be a concern. Therefore, no significant non-response bias or common method bias was detected in this study.

## Data Analysis and Results

### Descriptive Statistics

The descriptive statistics show a balanced sample of males (50.88%) and females (49.12%). Most respondents (59.73%) had an undergraduate or postgraduate qualification; most (82.74%) were over 31 years old, and almost all (96.90%) were non-contact and non-infectious for COVID-19. Overall, the descriptive analysis indicates that our sample is comprehensive and representative.

### Reliability and Validity

Cronbach’s α and Component Reliability (CR) were used to test the reliability of our survey instrument. As shown in Table 2, the Cronbach’s α and CR values of the main constructs are above 0.7 (between 0.895 and 0.969) indicating that the reliability of each variable is high (Fornell and Larcker, 1981). Secondly, in terms of construct validity, the factor loadings of each variable in the measurement model are all above 0.8. The Average Variance Extracted (AVE) values of the main constructs are all greater than 0.5 (see Table 1), which indicates good convergent validity. In addition, the square root of the AVE for each variable is greater than the correlation coefficient with all the other variables (Table 3). Therefore, the scale of this study has a high discriminant validity (Fornell and Larcker, 1981).

**Table 2:** Correlation coefficient and square root of AVE.

	GSM	KW	GA	GB	GI	PP	SEX	EDU	AGE
IQGSM	**0.931**								
KW	0.581	**0.932**							
GA	0.210	0.242	**0.914**						
GB	0.267	0.347	0.743	**0.939**					
GI	0.276	0.343	0.705	0.825	**0.927**				
PP	−0.094	−0.128	−0.160	−0.176	−0.172	**0.871**			
SEX	0.104	0.106	0.043	0.043	0.055	0.142	NA		
EDU	0.016	0.059	−0.236	−0.157	−0.206	0.065	0.085	NA	
AGE	0.015	0.082	−0.057	−0.052	−0.069	0.077	−0.161	0.075	NA

*The bold and italicized numbers on the diagonal are the square roots of AVE; GA, GB, and GI are first-order variables of local government trust; thus, the correlation coefficient is relatively high*.

**Table 3:** Bootstrapping analysis of the mediating effects.

The mediation path	Estimated value	95% Confidence interval	
		Percentile (Deviation correction)	
		Lower (2.5%)	Upper (97.5%)
Information Quality of GSM➔PPK➔PP	−0.049	−0.115	−0.005
Information Quality of GSM➔LGT➔PP	−0.041	−0.082	0.014
Information Quality of GSM➔LGT➔PPK➔PP	−0.005	−0.016	−0.002

Research suggests that government trust fits a “reflective-reflective” second-order model. Our data support this. Findings from our study indicate that local government trust fits a reflective-reflective second-order model. Firstly, the correlations between ability, benevolence, and integrity are all greater than 0.7. Secondly, the explanatory power (R^2^) of local government trust is greater than 80%. Lastly, the model indicates that local government trust has a strong impact on ability, benevolence, and integrity. The coefficients of those three dimensions (i.e., sub-variables) are all greater than 0.8 (*p* < 0.001). Therefore, it is reasonable to use the second-order “reflective-reflective” model to measure local government trust (Guo et al., 2016).

Ability, benevolence, and integrity are the three first-order reflection sub-variables of local government trust. Correlations between other variables are all below 0.6 (shown in Table 2) and all Variance Inflation Factors are around 2, which indicates that there is no serious collinearity among the four main variables.

### Hypotheses Testing

**Figure 2:**
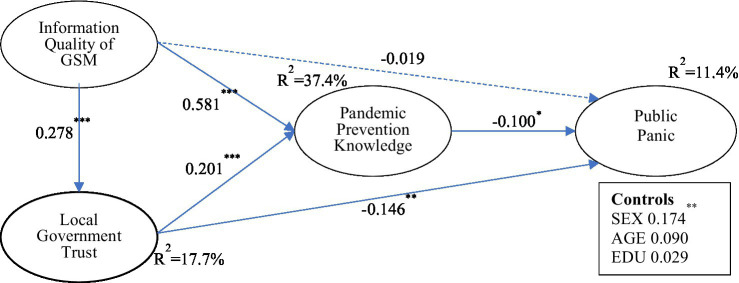
The model results. ^*^p < 0.10; ^**^p < 0.01; and ^***^p < 0.001. Local Government Trust is a second-order measurement model.

In this paper, Partial Least Squares Structural Equation Modeling (PLS-SEM) is used for structural model analysis and hypothesis testing. Since estimates of traditional PLS-SEM may be subject to inconsistency, researchers have developed a consistent PLS (PLSC) in recent years (Dijkstra and Henseler, 2015). This estimation method uses asymptotically normal estimators. The calculation method of PLSC is Two-Stage Least Square (2SLS). Smart PLS 3.0 software is employed for data analysis in our study. To ensure the consistency of estimations, the consistent PLS estimation algorithm and consistent PLS bootstrapping are used to test our research model (bootstrapping, *N* = 3,000). The results of the path analysis are shown in Figure 2. The SRMR index value of the overall model is 0.091, indicating a good fit (Henseler et al., 2015).

The results show that the Information Quality of GSM has a small and statistically insignificant (β = − 0.019; *p* > 0.10) negative on levels of public panic, so H1 is not supported. However, the Information Quality of GSM significantly and positively affects Pandemic Prevention Knowledge (*β* = 0.581; *p* < 0.001) and Pandemic Prevention Knowledge negatively affects Public Panic (*β* = −0.100; *p* < 0.10), which supports both H3 and H5. In addition, Information Quality of GSM has a significant positive impact on Local Government Trust (*β* = 0.278; *p* < 0.001) and Local Government Trust has a significant negative impact on Public Panic (*β* = −0.146; *p* < 0.01), supporting H6 and H2. Local Government Trust also has a significant positive impact on Pandemic Prevention Knowledge (β = 0.201; *p* < 0.001), so H4 is also supported. The sex of respondents also proved to have a significant factor impact on Public Panic, with females being more likely to panic. The other two control variables (Age and Education) were not significant.

Finally, we used Smart PLS’s blindfolding procedure to analyze the predictive correlations of the main dependent variables. The results show that the Stone-Geisser Q^2^ of Pandemic Prevention Knowledge, Local Government Trust, and Public Panic are 0.290, 0.054, and 0.051, respectively. All of them are greater than 0, so the results of this paper satisfy predictive validity (Peng and Lai, 2012).

### *Post-hoc* Mediation Analysis

To provide more insights, this section further tests the mediating effects of our model. Consistent bootstrapping is used to test the mediating roles of Pandemic Prevention Knowledge and Local Government Trust (resampling = 3,000). The results of the mediation analysis are shown in Table 3. Pandemic Prevention Knowledge was found to significantly mediate the effect between Information Quality of GSM and Public Panic (Information Quality of GSM ➔ PPK ➔ PP). Local Government Trust and Pandemic Prevention Knowledge together significantly mediate the effect between Information Quality of GSM and Public Panic (Information Quality of GSM ➔ LGT ➔ PPK ➔ PP). Surprisingly, Local Government Trust does not, on its own, significantly mediate the effect between Information Quality of GSM and Public Panic (Information Quality of GSM ➔ LGT ➔ PP). In conclusion, the results show that only Pandemic Prevention Knowledge plays a key mediating role between Information Quality of GSM and Public Panic.

## Discussion and Conclusion

Based on the theoretical framework of ELM, this study has investigated how local government can use social media to reduce public panic caused by the infodemic that arose alongside the COVID-19 pandemic. ELM proposes that persuasion occurs over two major routes: the central and the peripheral route. Reduction of public panic was found to occur mainly through argument quality (Information Quality of GSM) as the central route and source credibility (Local Government Trust) as the peripheral route. The main findings of our study are as follows. Firstly, the information quality of GSM contributes to pandemic prevention knowledge which, in turn, negatively affects public panic. Secondly, the information quality of GSM has a significantly positive impact on local government trust, and local government trust has a significant negative impact on public panic. Local government trust was also found to have a significant positive impact on pandemic prevention knowledge. Unfortunately, our study did not support the view that the information quality of GSM has a direct negative effect on public panic.

### Main Findings

#### Both Quality of Information on GSM and Local Government Trust Positively Affect Public Panic

Although the quality of GSM information has no significant direct effect on public panic, it is negatively correlated, suggesting that it may have a slight dampening effect. This may be due to its impact on pandemic prevention knowledge, which is a critical mediator between the quality of GSM information and public panic. Although some research has suggested that social media may adversely affect the public’s physical and mental health (McGowan et al., 2012; Basch et al., 2020; Laato et al., 2020), access to high-quality information (including epidemic data and refutation of inaccurate rumors) through GSM appears to stabilize public sentiment and alleviate panic. The results of this study also suggest that higher levels of public trust in the government are associated with reduced levels of panic caused by the infodemic. In the ELM, attitudes and responses to source credibility affect the attitudes and behaviors of people receiving information from that source. This peripheral route to persuasion is mainly based on perceptions of cues associated with the information, rather than the information itself (Petty and Cacioppo, 1986, 2012; Sussman and Siegal, 2003). Therefore, trust in local government as a credible source is important in reducing public anxiety, panic, and other negative emotions associated with the infodemic.

#### Pandemic Prevention Knowledge Is a Critical Mediator

This study also finds that pandemic prevention knowledge plays an important role in the mediating effect of GSM information quality on public panic. Firstly, the information quality of GSM has a significant impact on the improvement of public knowledge of pandemic prevention, suggesting that, by promoting relevant, high-quality information on social media, governments can transform it into knowledge that is useful for pandemic prevention. These findings are consistent with other ELM-based studies (e.g., Sussman and Siegal, 2003; Bhattacherjee and Sanford, 2006). Secondly, local government trust has a positive effect on pandemic prevention knowledge. In the information adoption model, source credibility often affects receivers’ perceptions of information usefulness (Sussman and Siegal, 2003). This paper further supports the view that trust in local government trust has a role in helping the public to transform received information into the knowledge of pandemic prevention. A low level of local government trust, by contrast, has been found to reduce the chance that the public will take precautions appropriate to the prevention and control of disease (e.g., Blair et al., 2017). Thirdly, the public’s knowledge level of pandemic prevention measures appears to have a negative effect on levels of infodemic-induced panic. This suggests that high-quality information from government social media can help the public to learn ways of preventing and alleviating the pandemic. The resulting improvement in pandemic prevention knowledge can enhance the public’s confidence in their ability to combat the pandemic and can help them to screen out misinformation. Both of these factors can help to reduce public panic and anxiety. Basch et al. (2020) also found that appropriate use of Tiktok can help the public to grasp health information and maintain their physical and mental health. Therefore, consistent with the prior information adoption model (Sussman and Siegal, 2003), the positive effect of local government trust on pandemic prevention knowledge provides further evidence for the critical mediating role of information usefulness (i.e., pandemic prevention knowledge) in changing users’ attitudes and emotions.

#### Information Quality of GSM Positively Effect on the Local Government Trust

By extending the original ELM, this paper supports the view that the quality of GSM information has a significant impact on public trust in government. The public learned about their government’s competence, benevolence, and integrity during the COVID-19 outbreak through government social media. When this information was perceived as complete, consistent, and accurate, public trust in the government was promoted. Earlier research also found that government social media can promote interactions between the public and the government, and help the public to better understand the government’s ability to manage emergencies (Zhang et al., 2019; Chen et al., 2020). Therefore, this paper complements the earlier ELM-based literature by exploring the relationship between argument quality and source credibility (Sussman and Siegal, 2003).

### Theoretical and Practical Implications

Using the framework of the Elaboration Likelihood Model, this paper has explored some of how government social media has influenced public panic during the COVID-19 pandemic. Our findings complement earlier research that explored the effects of social media in general during a pandemic and concluded inconsistent results of social media’s impacts during an infodemic (Basch et al., 2020; Laato et al., 2020). In addition, this paper has extended the scope of the ELM by applying it to public information behaviors in a pandemic situation. It has thrown light on the specific processes by which information dissemination through social media can improve the public’s mental health (through, for example, reducing panic). Therefore, our findings help to explain how the local government alleviates the negative effects of an infodemic through its social media, contributing to the research literature related to information epidemiology and government social media (Eysenbach, 2002, 2009; Mavragani, 2020). Last but not least, this paper draws on first-hand data collected during the first three weeks of the Wuhan’s lockdown at the start of the COVID-19 lockdown, to provide empirical evidence of value in directing the management of infodemics and reducing their negative impact. Also, in this Omnimedia era, it emphasizes the importance of the dissemination of reliable information and trust in local government in pandemic prevention and control. Specific recommendations arising from this research include the following. In a city lockdown, the government should make full use of social media to advise the public, providing them with knowledge relevant to pandemic prevention and helping them to identify and screen misinformation. Such actions can help to reduce the negative impacts of an infodemic. Appropriate use of GSM can also build public trust in their government, helping to assure them of its competence, and that it is acting for the public good. Finally, the public should actively participate in the government’s social media to improve their pandemic prevention knowledge and to remain up-to-date with their government’s pandemic control strategy. Together, this will promote a more cohesive response to the infodemic.

### Limitations and Further Directions

One clear limitation of this study is that it only explores how the government’s use of social media can reduce the negative impact of an infodemic. Chisita Collence (2020) states that academic, medical, and public librarians should mobilize their knowledge and skills to provide practical solutions to the public. Thus, future research may consider how librarians and other information professionals could use social media to support governments during an infodemic. Another useful direction for research arising from the findings of this paper is the influencing routes associated with the ELM. This paper only focuses on information quality as the central route and source credibility as the peripheral route; further studies may usefully explore other central and peripheral variables. Lastly, this paper reports on an analysis of survey data collected in Wuhan shortly after a lockdown was announced. Further research may adopt information epidemiology research methods (Mavragani, 2020) and take advantage of other Internet data (such as Weibo, WeChat, and Baidu.com) to identify and analyze the influencing mechanism of social media on information dissemination and information epidemic.

## Data Availability Statement

The raw data supporting the conclusions of this article will be made available by the authors, without undue reservation.

## Ethics Statement

The studies involving human participants were reviewed and approved by Central China Normal University. The patients/participants provided their written informed consent to participate in this study.

## Author Contributions

SZ and MC contributed to conception and design of the study. SZ organized the database, performed the statistical analysis, and wrote the first draft of the manuscript. SZ, YL, and MC wrote sections of the manuscript. All authors contributed to the article and approved the submitted version.

## Funding

The study was supported by The Young Top-notch Talent Cultivation Program of Hubei Province.

## Conflict of Interest

The authors declare that the research was conducted in the absence of any commercial or financial relationships that could be construed as a potential conflict of interest.

## Publisher’s Note

All claims expressed in this article are solely those of the authors and do not necessarily represent those of their affiliated organizations, or those of the publisher, the editors and the reviewers. Any product that may be evaluated in this article, or claim that may be made by its manufacturer, is not guaranteed or endorsed by the publisher.

## Footnotes

^1^ https://www.who.int/director-general/speeches/detail/munich-security-conference

^2^The concept of cyberchondria refers to the anxiety-amplifying effects of online health-related searches.
